# Mitochondrial Physiology and Gene Expression Analyses Reveal Metabolic and Translational Dysregulation in Oocyte-Induced Somatic Nuclear Reprogramming

**DOI:** 10.1371/journal.pone.0036850

**Published:** 2012-06-05

**Authors:** Telma C. Esteves, Olympia E. Psathaki, Martin J. Pfeiffer, Sebastian T. Balbach, Dagmar Zeuschner, Hiroshi Shitara, Hiromichi Yonekawa, Marcin Siatkowski, Georg Fuellen, Michele Boiani

**Affiliations:** 1 Max-Planck Institute for Molecular Biomedicine, Münster, Germany; 2 Laboratory for Transgenic Technology, Tokyo Metropolitan Institute of Medical Science, Tokyo, Japan; 3 Graduate School of Life and Environmental Sciences, University of Tsukuba, Ibaraki, Japan; 4 German Center for Neurodegenerative Disorders, Rostock, Germany; 5 Institute for Biostatistics and Informatics in Medicine and Ageing Research, University of Rostock, Rostock, Germany; Centro Cardiologico Monzino, Italy

## Abstract

While reprogramming a foreign nucleus after somatic cell nuclear transfer (SCNT), the enucleated oocyte (ooplasm) must signal that biomass and cellular requirements changed compared to the nucleus donor cell. Using cells expressing nuclear-encoded but mitochondria-targeted EGFP, a strategy was developed to directly distinguish maternal and embryonic products, testing ooplasm demands on transcriptional and post-transcriptional activity during reprogramming. Specifically, we compared transcript and protein levels for EGFP and other products in pre-implantation SCNT embryos, side-by-side to fertilized controls (embryos produced from the same oocyte pool, by intracytoplasmic injection of sperm containing the EGFP transgene). We observed that while EGFP transcript abundance is not different, protein levels are significantly lower in SCNT compared to fertilized blastocysts. This was not observed for Gapdh and Actb, whose protein reflected mRNA. This transcript-protein relationship indicates that the somatic nucleus can keep up with ooplasm transcript demands, whilst transcription and translation mismatch occurs after SCNT for certain mRNAs. We further detected metabolic disturbances after SCNT, suggesting a place among forces regulating post-transcriptional changes during reprogramming. Our observations ascribe oocyte-induced reprogramming with previously unsuspected regulatory dimensions, in that presence of functional proteins may no longer be inferred from mRNA, but rather depend on post-transcriptional regulation possibly modulated through metabolism.

## Introduction

Oocyte-induced reprogramming after somatic cell nuclear transfer (SCNT) affords unparalleled insight into the molecular mechanisms that enable nuclei to go from the differentiated to the pluripotent state. Within hours of SCNT, the ooplasm (i.e. the oocyte once deprived of its chromatin) confers the nucleus transplant epigenetic changes that can lead to an embryo-like gene expression pattern. To this end, gene expression must change both qualitatively and quantitatively (reprogramming). Specifically, most tissue-specific genes should become silenced – “somatic memory” erasure; [1], – embryonic genes activated [2], and housekeeping genes should meet the new host cytoplasm demands.

As somatic nuclei (e.g. from cumulus cells) are brought to the ooplasm, the cytoplasm-to-nucleus ratio changes dramatically, becoming unusually high compared to that in somatic cells [3]. Size can determine the capacity of a cell to synthesize and accumulate ubiquitous macromolecules such as ribonucleoproteins, in a mechanism known as “cell size regulation”. While expression level of many genes is dependent on cell size (e.g. ribosomal genes), that of ubiquitous transcription factors (like Oct1 and NF-Y) is not [3]. In SCNT embryos, mRNA levels are lower compared to fertilized controls during early cleavages [2], [4], while protein levels are poorly characterized. In this context, it is reasonable to hypothesize that after SCNT, the ooplasmic environment signals the nucleus, so that mRNA and protein levels can be adjusted accordingly.

A central open issue pertains as to which sensing mechanisms signal the change in cellular requirements to the nucleus, following SCNT. Metabolic regulatory networks in the ooplasm are likely to take part on the sensing/regulatory mechanisms determining the impact of cytoplasm-to-nucleus ratio changes. For example, mechanisms could include changes in intracellular reduction-oxidation (redox) state, which modulate gene expression by affecting transcription factor DNA binding capacity and activity [5]–[7], and stress-induced mitochondrial signalling operating through Ca^2+^ homeostasis alteration – i.e. retrograde response – which also results in gene expression change [8]. Furthermore, metabolism can act at the post-transcriptional level, for example in response to stimuli such as stress, leading to global translation inhibition [9]–[11].

In this study, we hypothesized that demands of a larger and different cytoplasm – the ooplasm – modulate the regulation of somatic nuclear activities in quantitative terms, with potential impact on the outcome of reprogramming. To test this hypothesis, we performed mouse SCNT with cells that harbour an actin promoter-driven *Cox8-EGFP* transgene [12]. *Cox8-EGFP* is transcribed in the nucleus, and the product is specifically localized to mitochondria. This experimental setting allows tracing of an embryonic-derived product, and thereby direct determination of how the somatic nucleus keeps up with host ooplasm demands after SCNT. Our data show that while transcripts of *Cox8-EGFP* achieve similar levels in SCNT as in ICSI-fertilized embryos, corresponding protein is lower in SCNT. However, for housekeeping genes such as *Gapdh* and *Actb* (maternal- and embryonic-derived), protein level differences reflected relative mRNA content. Following the characterization of metabolism after SCNT, we identified metabolic imbalances preceding the reported mRNA-protein mismatch, placing them as likely regulatory forces leading to the observed post-transcriptional changes in oocyte-induced reprogramming.

## Results

### Nuclear Reprogramming Visualization with Cox8-EGFP

The majority of conventional gene expression analysis methods cannot resolve between pre-existing (i.e. maternal) transcripts and those encoded by the transferred nucleus (i.e. somatic reprogramming product). A transgenic approach was chosen to selectively pursue the nucleus-encoded product (mRNA or/and protein) and thereby determine dynamics of nuclear reprogramming.

We used the Riken Institute mouse strain C57BL/6J-Tg(CAG-Cox8/EGFP)49Rin (here named briefly mtGFP-Tg mice), in which a transgene containing the transit peptide sequence of cytochrome c oxidase subunit VIII (Cox8) is fused to that of enhanced green fluorescent protein (EGFP), under the chicken beta-actin promoter [12]. As Cox8 is a nuclear-encoded but mitochondria-targeted protein, mitochondria in these mice are labeled with EGFP, allowing live non-invasive visualization of mitochondria, as shown previously in various tissues [13].

**Figure 1:**
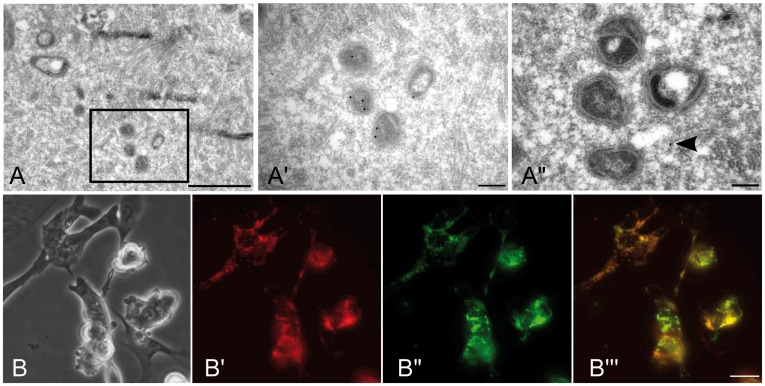
EGFP localization in mtGFP-Tg cells. (A) In the mtGFP-Tg oocyte, EGFP is mainly localized in mitochondria (A’, insert in A), as shown by cryo-immuno electron microscopy. (A”) In cryosections of B6C3F1 wild-type ooplast (SCNT recipient), only low unspecific background labeling in the cytoplasm is found (arrow), while there is no detectable mitochondrial signal. Scale bar, A: 1 µm; A’ and A”: 200 nm. (B) Embryonic stem cells (here feeder-free) derived from fertilized blastocysts express EGFP (B”, green), co-localizing with mitochondria (stained with TMRM; B’, red); B’”, merge B’ and B”. Scale bar, 25 µm.

Localization of EGFP to mitochondria – a key precondition to use the Cox8-EGFP construct to study nucleus-cytoplasmic interaction during reprogramming – was shown by cryo-immuno electron microscopy, where EGFP was detected by anti-GFP antibody labeled by protein A gold. The signal is found specifically in the mitochondrial membranes and matrix of the mtGFP-Tg oocyte (**Figure 1A–A”**). Furthermore, mitochondria-specific EGFP labeling was confirmed in embryonic stem cells (ESCs). Specifically, ESCs mitochondria were labeled with tetramethylrhodamine methyl ester (TMRM) and imaged simultaneously for EGFP (**Figure 1B’-B”**). As previously shown for other cell types [13], not all cells showed expression of EGFP; yet, all detected EGFP co-localized to mitochondria (**Figure 1B’”**).

**Figure 2:**
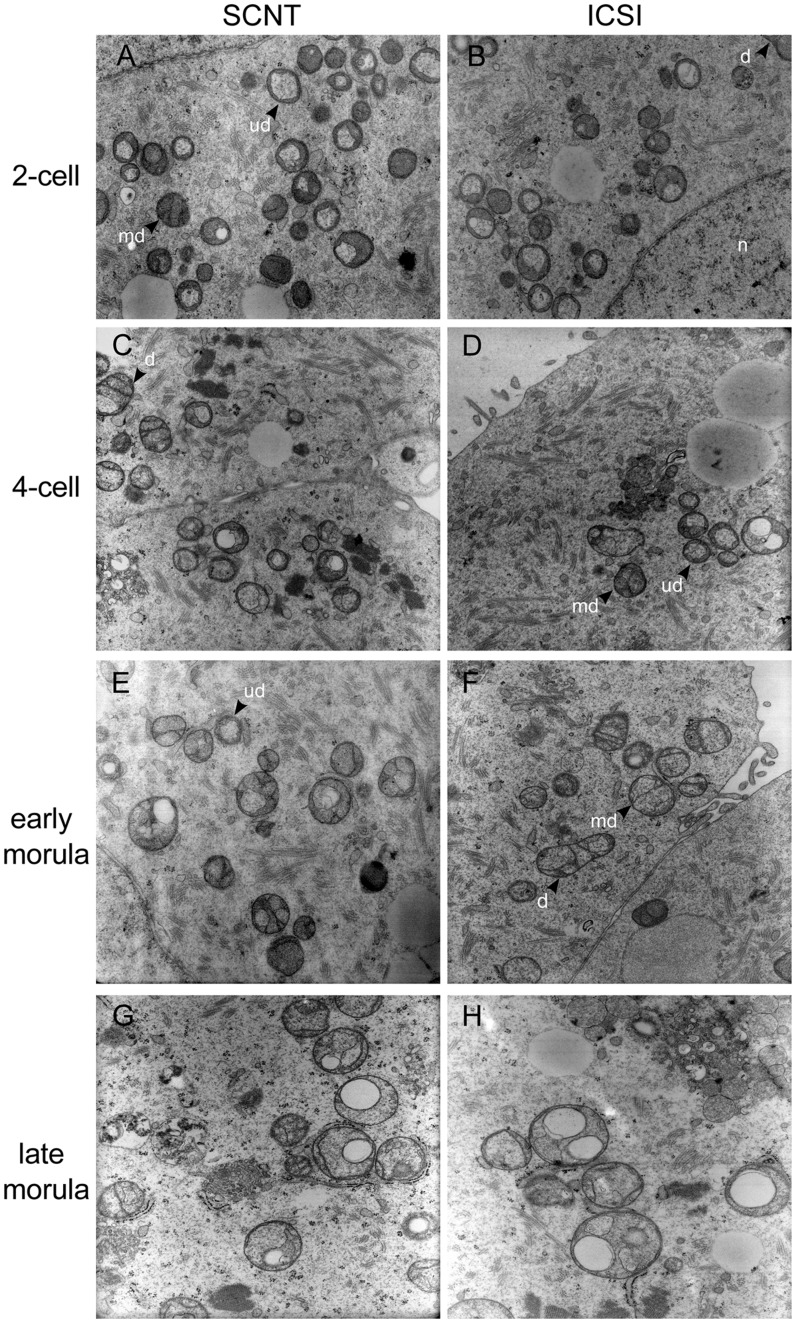
Mitochondria ultrastructure in pre-implantation embryos. Representative micrographs show mitochondria ultrastructure in mouse wild-type SCNT and ICSI embryos, throughout pre-implantation development. nucleus: n; mitochondria: d, developed; md, medium developed; ud, undeveloped. Mitochondria morphology and analysis are described in Materials and Methods. Magnification: 12200X.

Due to close proximity to the nucleus and nature of micromanipulation procedure, some donor mitochondria from mtGFP-Tg cumulus cells are likely to be carried along with the nucleus during SCNT into wild-type ooplasts. Transmission electron microscopy (TEM) analysis based on mitochondria morphology in ultrathin sections of periphery, perinuclear and nuclear regions in early pronuclear stage wild-type SCNT embryos could not identify cumulus cell-like mitochondria (data not shown). Furthermore, analysis throughout pre-implantation development showed high similarity in mitochondria morphology and maturation between SCNT and ICSI (**Table 1**; **Figure 2**, for representative embryonic mitochondria morphology).

**Table 1:** Ultrastructural analysis of mitochondria in pre-implantation embryos.

	mitochondria global morphology				mitochondria cristae morphology					
	round		elongated		Developed		medium		undeveloped	
stage	ICSI	SCNT	ICSI	SCNT	ICSI	SCNT	ICSI	SCNT	ICSI	SCNT
**2-cell**	20.6±3.0	21.3±7.9	0.7±0.2	0.6±0.02	3.6±1.8	6.2±6.5	12.0±3.3	10.4±7.8	5.3±2.3	4.8±4.1
**4-cell**	14.4±1.6	12.2±1.6	0.6±0.6	1.2±0.5	0.6±0.7	2.0±0.6	6.4±0.5	6.7±0.5	8.8±5.2	4.6±0.5
**morula**	13.1±1.3	13.9±0.5	0.5±0.7	0.3±0.3	1.1±0.7	0.4±0.2	5.8±0.9	6.4±1.0	6.8±2.0	8.2±0.1

Mitochondria morphology was analyzed in early (2- and 4-cell) and later stage (morula) SCNT and ICSI embryos. Round and elongated mitochondria were counted per section (nuclear or perinuclear region) of a single blastomere (2–3 blastomeres per embryo, n = 3; 3000X). Numbers are mean (± standard deviation) of analyzed areas. Mitochondrial cristae morphology (developed, medium developed and undeveloped; see also Figure 2) was characterized to define level of mitochondrial maturation.

Based on these observations, we conclude that Cox8-EGFP is specifically targeted to mitochondria, and that EGFP detected after cumulus cell nuclear transfer to the ooplasm is encoded by the somatic nucleus, i.e. is the product of nuclear reprogramming and not of extended donor cell mitochondria carryover due to micromanipulation.

### Lower Cox8-EGFP in SCNT Compared to Fertilized Embryos

**Figure 3:**
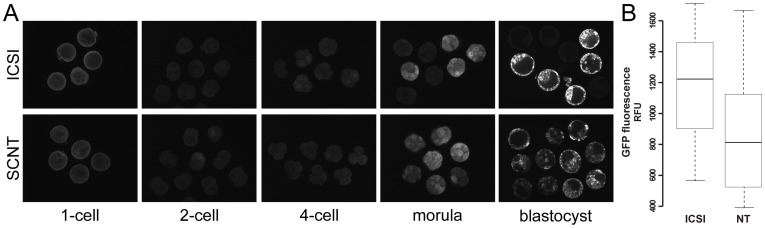
EGFP intensity in mtGFP-Tg embryos. B6C3F1 (wild-type) oocytes and ooplasts were used as recipients for mtGFP-Tg sperm or cumulus cell, for ICSI and SCNT, respectively. (A) Immunocytochemistry in ICSI and SCNT pre-implantation embryos revealed GFP expression onset at morula stage, and higher levels of GFP in ICSI compared to SCNT blastocysts. Hemizygosity of mtGFP-Tg mice results in EGFP-positive and negative ICSI embryos, while all SCNT embryos derive from EGFP-positive cumulus cell donors. (B) EGFP-specific signal quantification in mtGFP-Tg blastocysts confirmed higher EGFP protein levels in ICSI embryos (t-test, *p* = 0.006). Box plot: fluorescence intensities distribution in the blastocyst; top and bottom lines: inter-quartile range; middle line: median; whiskers: range of variation limited to 1.5 times inter-quartile range.

**Figure 4:**
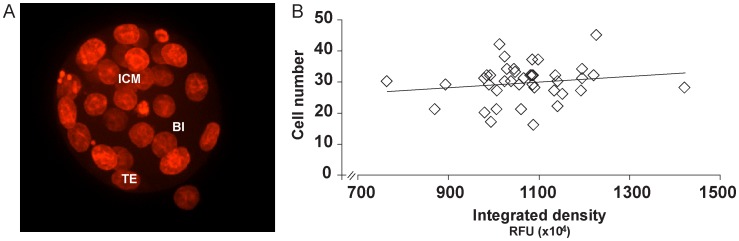
EGFP intensity and embryo cell number. (A) Fertilized (B6C3F1×mtGFP-Tg) blastocysts were stained with DRAQ5 to assess total cell number. ICM: inner cell mass; Bl: blastocoel; TE: trophectoderm. (B) Total cell count shows low correlation (r^2^ = 0.027) with EGFP levels. Each diamond corresponds to one embryo.

Sperm or cumulus cells nuclei from mtGFP-Tg mice were used to perform ICSI and SCNT, respectively, into wild-type (B6C3F1) recipient ooplasts, in order to distinguish between products of maternal or embryonic origin. Here, the traced Cox8-EGFP is always the product of the paternal allele, as the SCNT cumulus cell donors were daughters of the ICSI sperm donors. As during live imaging, early cleavage embryos have high autofluorescence in the EGFP emission range – namely 488 nm, due to mitochondria-located reducing equivalent FAD^2+^; data not shown [14], – a red fluorochrome-tagged antibody against GFP was used to trace nuclear-encoded transgene product onset (**Figure 3A**). The immunofluorescent signal was detected earlier in ICSI compared to SCNT, starting with weak 4-cell stage nuclear signal. This became stronger in the morula in both SCNT and ICSI embryos, coinciding with the onset of mitochondrial DNA (mtDNA) replication and transcription that prepares the embryo for implantation and post-implantation development [15]. In the blastocyst, immunofluorescent signal increased for ICSI, whereas SCNT showed lower signal (**Figure 3A**). This observation was confirmed through live EGFP confocal imaging of SCNT and ICSI blastocysts 3.5 days after activation (**Figure 3B**). Through total cell count using nuclear stain DRAQ5 (**Figure 4A**), we determined that mtGFP-Tg SCNT and ICSI blastocysts have no significant difference in cell numbers (SCNT: 44.7±12.1, n = 7; ICSI: 41.9±13.3, n = 15; expressed as mean ± standard deviation). While previously, cumulus cells SCNT gave rise to embryos with lower cell numbers compared to ICSI [16], the present observation indicates that in our setup, higher or lower EGFP intensity is independent of cell number.

Furthermore, we determined whether EGFP resulted in toxicity for the embryo. Blastocyst cell number showed no correlation with EGFP mean intensity, as demonstrated for fertilized embryos (**Figure 4B**). Also, comparative TEM analysis of defined regions of late pre-implantation SCNT and ICSI embryos shows no difference in mitochondria number (**Table 1**).

While confirming the mtGFP-Tg transgenic model suitability for nucleus-encoded products visualization, discerning these from preexisting maternal protein, our observations indicate disturbances in gene and/or protein regulation in reprogramming after SCNT.

### EGFP mRNA does not Reflect Protein in mtGFP-Tg Blastocysts

**Figure 5:**
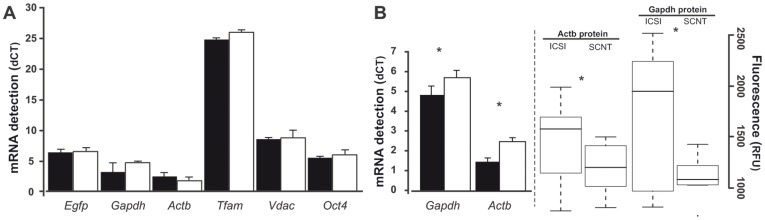
Transcription and translation in mouse blastocysts. mRNA levels were determined for pools of six ICSI and SCNT blastocysts (in duplicate), by quantitative RT-PCR, over two independent experiments. Technical replicates were determined three times for each sample, and results were normalized to Flag-Oct4 mRNA. (A) dCT values for transcripts in mtGFP-Tg SCNT, compared to ICSI. (B) dCT values for Gapdh and Actb mRNA, as well as protein levels (by immunocytochemistry), in wild-type SCNT relative to ICSI. Gapdh and Actb levels were significantly different, both for mRNA (t-test, *p* = 0,001 and *p* = 1,29E-08, respectively) and protein (t-test, *p* = 0.006 and *p* = 0.04, respectively). Box plot: fluorescence intensities distribution in the blastocyst; top and bottom lines: inter-quartile range; middle line: median; whiskers: range of variation limited to 1.5 times inter-quartile range.

We aimed to determine whether EGFP differences in SCNT and ICSI blastocysts resulted from problems arising at transcriptional or post-transcriptional level. Quantitative RT-PCR was performed on pools of EGFP-positive blastocysts. Importantly, an external reference mRNA was added to the samples and used for transcript level normalization [4]. This is an important aspect, as levels of genes generally used as housekeepers show high variance during normal [17], [18] and SCNT [18] mouse pre-implantation development. Results (shown in **Figure 5A**) revealed that mtGFP-Tg SCNT and ICSI blastocysts contained similar EGFP mRNA amounts, indicating that lower protein in SCNT blastocysts is not due to lower transcript. We further analyzed how mRNA levels compared for genes other than *Cox8-EGFP*, in ICSI and SCNT blastocysts. mRNA content for *Gapdh* and *Actb* (generally considered housekeeping genes) showed a small but not significantly different increase in SCNT compared to ICSI. The pluripotency marker *Pou5f1* (also known as Oct4) showed only a small mRNA decrease in SCNT embryos. Also mRNA levels for mitochondria transcription factor *Tfam* and mitochondrial structural protein Voltage-dependent anion channel (*Vdac*), both nuclear-encoded, showed no significant difference. These observations suggest that after mtGFP-Tg SCNT, reprogramming allows appropriate mRNA regulation of genes including housekeepers, and genes encoding transcription factors and structural proteins. This indicates that the transferred nucleus likely meets most of the larger transcript level demands of the ooplasm, while synthesis of certain proteins is misregulated.

### No mRNA-protein Mismatch for Gapdh and Actb after SCNT

We aimed to determine whether gene products other than Cox8-EGFP would also experience transcript-protein imbalance after SCNT. Embryos used for this analysis were congenic with the mtGFP-Tg embryos; that is, these embryos are genetically identical, except for the Tg locus ([19]; see “Oocyte micromanipulation and embryo culture” in Materials and Methods section for details). We evaluated mRNA and protein levels for Gapdh and Actb (glycolytic and structural proteins, respectively), after SCNT and ICSI (B6C3F1 oocytes as recipients, B6C3F1 cumulus cells for SCNT, and C3H males sperm for ICSI). Quantitative RT-PCR revealed that both *Gapdh* and *Actb* transcript levels did not vary significantly, although they were lower in SCNT relative to ICSI (**Figure 5B**). Interestingly, immunocytochemistry for Gapdh and Actb showed that protein levels were also lower in SCNT blastocysts compared to ICSI (**Figure 5B**). Together, results indicate that post-transcriptional dysregulation after SCNT does not apply to all gene classes.

### Metabolism-related mRNAs Affected upon SCNT

The observed imbalance between mRNA and protein level for certain gene products in SCNT embryos may stem from a deficit of mRNA translation into protein. This deficit could have multiple causes, such as ribosome turnover and mRNA binding to the ribosomes, both of which are subjected to metabolic regulation [10]. In this context, we performed a whole transcriptome analysis to assess the extent and nature of transcriptional changes in SCNT embryos compared to fertilized counterparts, with emphasis on gene functions related translation and metabolism.

Gene set enrichment analysis (GSEA) was performed on a list of 242 differentially expressed genes (up and downregulated) in wild-type SCNT (i.e. congenic to mtGFP-Tg) versus ICSI embryos at the 8-cell stage, to identify significantly overrepresented categories. Interestingly, results show that metabolism-related gene ontology (GO) terms are overrepresented, as well as terms related to cellular process regulation and cell communication (**Table 2**). This indicates that in our experimental setup, impaired translation of metabolism-related gene products may impact in cellular function during early stages of reprogramming after SCNT.

**Table 2:** Gene Ontology (GO) categories enriched among the genes that are differentially expressed (2-fold) in SCNT and ICSI embryos.

GO:ID	Term	Annotated	Significant	Expected	parentChild.Fisher
32501	multicellular organismal process	1485	71	44.35	1.30E-05
10604	positive regulation of macromoleculemetabolic process	632	33	18.87	3.30E-05
1775	cell activation	174	16	05. Feb	4.90E-05
32502	developmental process	1350	62	40.32	0.00019
8219	cell death	668	36	19.95	0.00021
50794	regulation of cellular process	2895	109	86.46	0.00029
16265	death	670	36	20. Jan	0.00035
9893	positive regulation of metabolic process	672	36	20. Jul	0.00045
71702	organic substance transport	155	13	Apr 63	0.00066
60255	regulation of macromolecule metabolic process	1709	64	51.04	0.00074
48519	negative regulation of biological process	1161	53	34.67	0.00076
10605	negative regulation of macromoleculemetabolic process	510	25	15.23	0.00086
65007	biological regulation	3199	119	95.54	0.00092
50789	regulation of biological process	3070	115	91.68	0.00094
46164	alcohol catabolic process	45	7	Jan 34	0.00098
32787	monocarboxylic acid metabolic process	145	14	Apr 33	0.00117
44275	cellular carbohydrate catabolic process	52	7	Jan 55	0.00121
6096	glycolysis	27	6	0.81	0.00127
42107	cytokine metabolic process	19	4	0.57	0.00131
48863	stem cell differentiation	34	6	01. Feb	0.00132
48523	negative regulation of cellular process	1082	48	32.31	0.00154
16052	carbohydrate catabolic process	58	7	Jan 73	0.00181
48522	positive regulation of cellular process	1180	51	35.24	0.0022
7154	cell communication	1335	56	39.87	0.00221
48518	positive regulation of biological process	1291	55	38.56	0.00298
2376	immune system process	374	21	Nov 17	0.00384
51247	positive regulation of protein metabolic process	214	14	Jun 39	0.00448
65009	regulation of molecular function	690	38	20.61	0.00483
31325	positive regulation of cellular metabolic process	644	31	19.23	0.00565
8283	cell proliferation	490	25	14.63	0.00579
10628	positive regulation of gene expression	415	21	Dez 39	0.00581
44259	multicellular organismal macromoleculemetabolic process	15	3	0.45	0.00628
31399	regulation of protein modification process	366	18	Okt 93	0.00671
6793	phosphorus metabolic process	808	35	24.13	0.00796
6066	alcohol metabolic process	221	13	06. Jun	0.00812
6897	endocytosis	153	8	Apr 57	0.00829

130 upregulated and 112 downregulated genes in SCNT compared to ICSI. *p*-values were truncated at 0.00001.

### Metabolic Disturbances Precede mRNA-protein Mismatch During Reprogramming

Post-transcriptional regulation in response to stimuli like stress and metabolic-repressive conditions may lead to translation inhibition [9]–[11], [20], [21]. It is then formally possible that post-transcriptional dysregulation in the late pre-implantation SCNT embryo derives from metabolic disturbances. We therefore aimed to determine whether metabolic regulation is affected during early reprogramming, and later in SCNT embryo pre-implantation development, when the EGFP mRNA-protein mismatch is observed.

**Figure 6:**
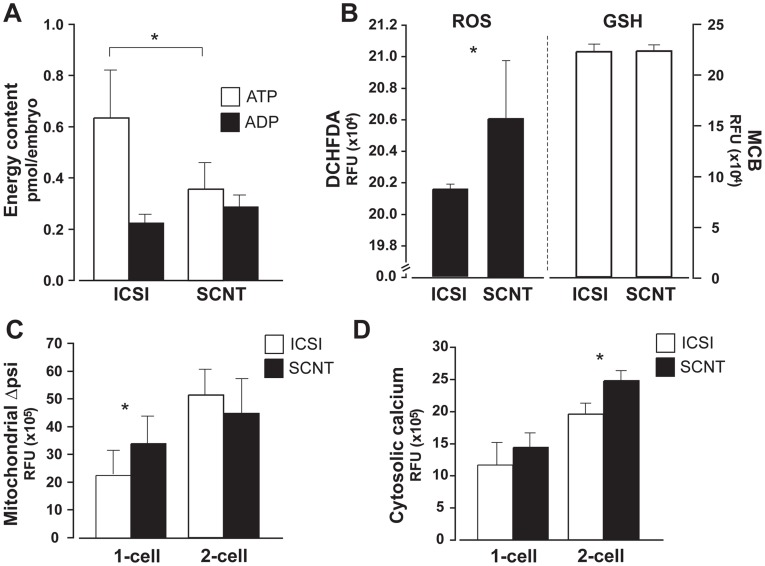
Metabolic disturbances after SCNT. (A) Energy (ATP and ADP) content decreases in SCNT at the 4-cell stage, compared to ICSI embryos (significant difference for ATP; t-test, *p* = 0.008). (B) Levels of reactive oxygen species (ROS) are increased in 4-cell SCNT (t-test, *p* = 1.0E-06), as measured by DCHFDA. At the same stage, cellular antioxidant capacity (measured as reduced glutathione levels, with MCB) is similar for SCNT and ICSI embryos. (C) Increased TMRM signal indicates higher mitochondrial membrane potential after SCNT (t-test, *p* = 0.007), while (D) cytosolic calcium (measured with Fura-2 AM) is significantly increased in 2-cell SCNT (t-test, *p* = 1.4E-06).

Due to the fluorimetric nature of some of the assays, and to avoid potential interference with EGFP, SCNT embryos were derived from B6C3F1 instead of mtGFP-Tg cumulus cell nuclei. ATP/ADP content were measured for SCNT and ICSI embryos during pre-implantation development. Energy capacity was constant throughout all stages (data not shown), with exception of 4-cell, when ATP levels were significantly lower for SCNT than ICSI (**Figure 6A**). Furthermore, oxidative stress indicators in the embryos were measured. Reactive oxygen species (ROS), determined using a probe specific for hydrogen peroxide and related peroxides (DCHFDA), were significantly elevated in SCNT compared to fertilized counterparts, at 2- (data not shown) and 4-cell (**Figure 6B**). This elevation was not accompanied by changes in reduced glutathione (**Figure 6B**), indicating reduced cellular antioxidant defence mechanisms in SCNT embryos.

As previously mentioned, retrograde response is launched as a reaction to mitochondrial stress. It starts with an alteration in mitochondrial membrane potential resulting in elevated cytosolic Ca^2+^ and consequent activation of Ca^2+^/calmodulin-responsive calcineurin and related factors, leading to upregulation of nuclear marker genes related to Ca^2+^ storage/transport [8]. We used the potentiometric dye TMRM to determine mitochondrial membrane potential during early cleavages after SCNT and ICSI. Results show a small elevation on membrane potential in the SCNT at the 1-cell stage, 6 hours after nuclear transfer (**Figure 6C**). This suggests the possibility of mitochondrial stress induced by the somatic nucleus presence, which could be responsible for higher ROS in 2-cell SCNT embryos. Furthermore, free cytosolic Ca^2+^ was also measured. Interestingly, we observed increased Ca^2+^ in 2-cell SCNT embryos compared to ICSI (**Figure 6D**), strongly suggesting that retrograde response is initiated during the initial stages of nuclear reprogramming.

As no significant changes in mitochondria morphology were detected via TEM between SCNT and ICSI embryos (**Table 1**), impact of energy and Ca^2+^ metabolism disturbances after SCNT is likely low at the (mitochondria) structural level. Together, results show that metabolic imbalances occur during initial cleavages after SCNT, i.e. initial somatic nuclear reprogramming, and precede the reported mRNA-protein mismatch.

## Discussion

Previous studies showed that a specific nucleus-to-cytoplasm ratio needs to be maintained upon nuclear transplantation, in order not to affect developmental capacity [22]. Reduced cytoplasm volume decreases oocyte’s reprogramming potential in bovine SCNT [23], [24], and additional recipient ooplasm results in the same negative effect [23], also in mouse [25]. In this study, we investigated how the somatic nucleus responds to the increased nucleus/cytoplasm ratio ensued after SCNT into mouse oocytes, and explored the underlying signalling mechanisms including energy metabolism, which is a crucial mediator of nucleus-cytoplasm communication.

By performing ICSI and SCNT using transgenic mtGFP-Tg cells [12] followed by non-invasive live-cell visualization [13], a strategy was developed to allow direct tracing of a nuclear-encoded product – discriminating between products of maternal and embryonic origin, – and its mobilization dynamics to a particular cytoplasmic sub-cellular compartment. Importantly, the transgenic approach used for selective pursuit of mRNA or/and protein allowed determination of onset and dynamics of transcriptional/post-transcriptional activity during oocyte-induced reprogramming. Our data show that while SCNT and ICSI blastocysts contain similar levels of EGFP transcript, protein levels are lower in SCNT. We further determined that the effect seen for EGFP – which is encoded by the somatic nucleus and thereby of embryonic origin – was not observed for two gene products of maternal and embryonic origin. Specifically, SCNT blastocysts showed lower Gapdh and Actb protein abundance relative to ICSI, while transcripts showed similar trend. Together, observations add a new, previously undervalued dimension – i.e. post-transcriptional regulation – to the understanding of oocyte-induced nuclear reprogramming.

Although the oocyte is a special cell regarding transcription and translation (as numerous transcripts are present but not translated until after fertilization), the question arises as to why for some genes, the SCNT embryo’s genome would be adequately reprogrammed at the mRNA but not at the protein level. Previous reports show that SCNT mouse embryos do not degrade maternal mRNAs as efficiently as fertilized counterparts [26]. In our experimental design, where the SCNT donor nucleus contains the transgene while the recipient ooplasm does not, EGFP mRNA is exclusively of embryonic origin. As maternal mRNA is therefore not present, the mechanism regulating *Cox8-EGFP* mRNA content or interfering with its translation into protein must be other than that described by Vassena and colleagues [26].

As a possibility, we considered that the SCNT embryo’s mRNAs may fail to be translated, as a consequence of the altered epigenetic status of the donor cell [27]. Underlying causes could involve ribosomal deficit (e.g. reduced turnover of ribosomal riboproteins), but also reduced recruitment and/or translation of mRNA to the polyribosomes (which is under the influence of metabolism and response to stress; [28]) and/or to low levels of ATP (as larger amounts of energy are required for translation, compared to transcription [29]). Interestingly, whole transcriptome analysis of SCNT and ICSI 8-cell stage embryos revealed that the gene ontology (GO) terms most represented among the differentially expressed genes are related to metabolism. It therefore follows that the abnormalities detected in metabolism-related transcription and translational machinery of SCNT embryos may impact on the embryo’s energy metabolism.

A link between metabolism, pluripotency and reprogramming has been suggested. A recent study based on metabolic and proteomics analyses indicates the requirement for the transition from somatic oxidative phosphorylation energetics into glycolysis, in order to fuel pluripotency after direct-induced somatic reprogramming [30]. In SCNT, a recent report showed reduced blastocyst development and pluripotent markers expression in fertilized mouse zygotes reconstructed to contain porcine cytoplast, suggesting that dysfunctional nucleus-cytoplasm communication contributes to the failure of inter-species SCNT [31]. Considering the wide range of critical roles of mitochondria, influencing cell function and embryonic development, disturbed mitochondria function in particular has been suggested to directly impact on SCNT reprogramming [32]. Abnormal (i.e. continuous) expression of the nuclear-encoded mitochondrial transcription and replication factors Tfam and PolG follows nuclear transfer by fusion in sheep, indicating aberrant mitochondria-nucleus cross-talk [33]. Here, we analysed pre-implantation SCNT mouse embryos to assess the level of oxidative stress and metabolic activity during/after initial nuclear reprogramming. As some of the relevant assays are fluorimetric, we analyzed SCNT and ICSI embryos derived from donor cells that have identical genetic background to mtGFP-Tg, but lack the transgene (i.e. to avoid interference of EGFP). Interestingly, data show global metabolic disturbance, detected as early as 6 hours post-activation. After a previous report showing dysregulation of stability – and possibly translation – of some maternal mRNAs at the first two cell cycles after SCNT [26], our study is the first to show that SCNT embryos are distinguishable from fertilized counterparts (at the metabolic/physiological level) already shortly after manipulation. These early differences could indicate different metabolic requirements, which could explain why early pronuclear stage (but not later stages) mouse SCNT embryos develop in culture, but arrest when transferred to the uterine environment [16]. Importantly, metabolic disturbances precede the onset of post-transcription dysregulation in mouse oocyte-induced reprogramming. Specifically, compared to ICSI, SCNT embryos experienced elevated mitochondrial membrane potential (at 1-cell), and elevated cytosolic Ca^2+^ (2-cell), followed by lower energy content and higher levels of oxidative stress (4-cell).

**Figure 7:**
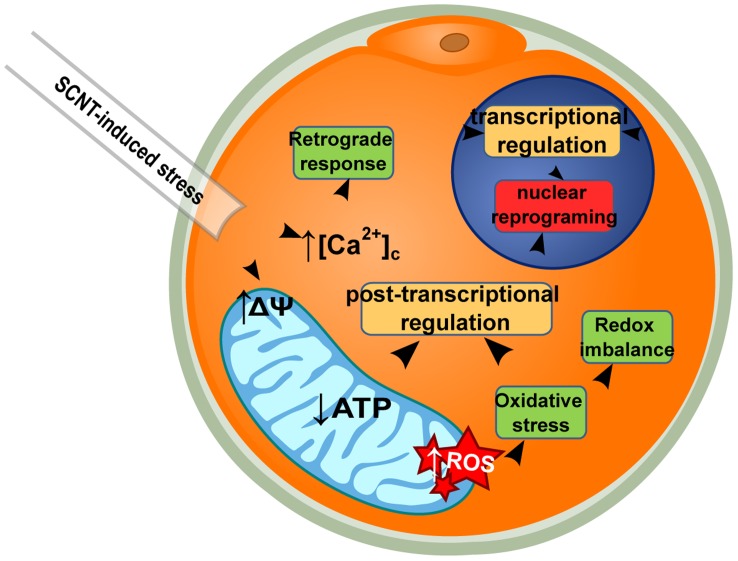
Model of post-transcriptional regulation after SCNT. Interlinked events at the metabolic level, disturbed after SCNT, are proposed to impact on reprogramming and embryo development outcome. SCNT manipulation alters mitochondrial membrane potential (Δψ), triggers cytosolic Ca^2+^ concentration ([Ca^2+^]_c_) increase, ATP decrease and reactive oxygen species (ROS) increase. Increased cytosolic Ca^2+^-induced retrograde response, and ROS-induced redox imbalance, could induce transcriptional regulation. Oxidative stress and decreased ATP could induce cellular responses driving post-transcriptional regulation. Proposed effects could be direct (bold arrows), or follow after a cascade of events (indirect; dashed arrows).

Observations strongly indicate that a series of interlinked events at the metabolic level is disturbed after SCNT, potentially with high impact on reprogramming and embryo development. We propose that SCNT triggers early mitochondrial membrane potential alterations, increase in oxidative stress and cytosolic Ca^2+^, and energy content decrease (**Figure 7**). Interestingly, such events are candidates to determine retrograde response onset, a mitochondria-to-nucleus communication pathway with direct impact on gene expression [**8**] that is regulated by the stress-sensitive sub-cellular localization of certain transcription factors [34]. As retrograde response is linked to the onset of embryonic genome activation [35], and nuclear reprogramming success in bovine SCNT benefits from tight regulation of Ca^2+^ oscillations [36], it is plausible that disturbed metabolism affects mouse SCNT embryo development directly. Importantly, metabolic disturbances could be direct causes for post-transcriptional dysregulation in the late pre-implantation SCNT embryo. The energy costs for the production of a functional protein are high, starting with translation initiation, which requires ATP [37]. Interestingly, reduction in cellular ATP derived from hypoxia-induced stress in *Arabidopsis* was reported in association to similar overall translational rates decay [28]. Other cellular responses to stress conserved among eukaryotes involve post-transcriptional regulation such as induction of pre-RNA alternative splicing, differential nuclear export, compartmentalization and degradation of mRNA, and modification of translation initiation factors, all with direct impact on mRNA stability and protein synthesis [9], [11]. Interestingly, previous work has shown that nuclear reprogramming success in bovine SCNT benefits from the correction of cytoplasmic Ca^2+^ increase resulting from SCNT-induced stress originated in the endoplasmic reticulum (ER) [38]. As ER-induced stress has been reported to exert translational control – via activation of the unfolded protein response and ROS production leading to apoptosis [39], also in pre-implantation mouse embryos [40], – it is formally possible that metabolic disturbances detected during oocyte-induced reprogramming trigger post-transcriptional processes leading to lower protein levels after SCNT. Interestingly, reprogramming-related metabolic changes develop without mitochondrial structural changes being established, as shown by TEM. This indicates a preferential direction in reprogramming mechanisms: the ooplasm preserves its structural character (i.e. oocytic mitochondria are not converted into somatic-like), as the somatic nucleus is reprogrammed to meet embryo’s requirements. In conclusion, we propose that metabolic disturbances detected very early after SCNT are regulatory forces leading to the observed post-transcriptional dysregulation in the context of mouse oocyte-induced nuclear reprogramming.

## Materials and Methods

### Ethics Statement

This study was performed in accordance with the Federation of European Laboratory Animal Science Associations (FELASA) recommendations. The protocol was approved by the Landesamt für Natur, Umwelt und Verbraucherschutz (LANUV) of the state of North Rhine-Westphalia, Germany (Permit Number: 87–51.04.2010.A160). Every effort was therefore made to minimize animal suffering.

### Animal Handling and Oocyte Retrieval

Oocytes from B6C3F1 (C57Bl/6J×C3H/HeN) mice were used as somatic nuclei recipients. Females were superovulated by injection of 10 international units (IU) of pregnant mare serum gonadotropin (Calbiochem EMD Biosciences, San Diego, CA, USA), and 10 IU of human chorionic gonadotropin (hCG; Calbiochem) 48 h later. Animals were sacrificed by cervical dislocation 14 h after hCG injection, and oocytes retrieved from oviduct ampullae. Oocytes/cumulus cells were manipulated in Hepes-buffered CZB (HCZB) medium, as described [41], [42]. Separation of cumulus cells from oocytes was done by treatment with 50 IU mL^-1^ hyaluronidase (ICN Biomedicals, Eschwege, Germany) in HCZB, and gentle pipetting. Cells were placed in embryo culture medium, i.e. alpha modified Eagle’s medium (α-MEM; M4526 Sigma, Germany) supplemented with 0.2% w/v BSA (Pentex; Serological Proteins Inc., Kankakee, IL, USA) and 50 µg mL^−1^ gentamicin sulfate (ICN Biomedicals), 37°C 5% CO_2_.

### Oocyte Micromanipulation and Embryo Culture

Micromanipulations and embryo culture were performed as previously described [41]–[43]. For SCNT, oocytic spindle-chromosome complex were removed (enucleation) before cumulus cell nucleus transfer, with a piezo actuator-driven micropipette under DIC optics. Cumulus cells were from 5 week-old oocyte donors, either from B6C3F1, or the F1 offspring derived from C57BL/6J-Tg(CAG-Cox8/EGFP)49Rin (common name mtGFP-Tg; RIKEN-BNL Research Center No. RBRC02250) crossed with C3H/HeN male; it follows that the F1 transgenic mice and the B6C3F1 mice are congenic. Reconstructed oocytes were activated for 6 h in calcium-free embryo culture medium supplemented with 10 mM SrCl_2_ (ICN) and 5 µg mL^−1^ cytochalasin B (Calbiochem). Intracytoplasmic sperm injection (ICSI) into intact B6C3F1 oocytes (same pool as SCNT) was used as fertilization control, using sperm from 5 week-old mice of C57Bl/6, or mtGFP-Tg (C3H/HeN background) males. Embryos were placed in embryo culture medium, 37°C 5% CO_2_.

### Embryonic Stem Cells (ESCs) Derivation

ESC lines derivation from blastocysts (obtained after in vitro culture of naturally fertilized oocytes, mtGFP-Tg C57Bl/6×129SV) was performed as described previously [42]. After zonae pellucidae removal with Tyrode’s solution (Sigma), blastocysts were transferred onto a confluent feeder layer of γ-ray inactivated mouse embryonic fibroblasts (C3H/HeN background), in 4-well dishes. Day 7 after fertilization, trophoblastic outgrowths were removed with polished glass pipette, trypsinized with 0.25% trypsin-EDTA (Gibco, Gaithersburg, MD, USA) and reseeded (96-wells). Dissociated ICMs were grown for 6 days, then expanded onto larger well sizes, by subsequent passaging. ESC culture media was composed of Knockout DMEM (Gibco) with 15% serum replacement (Gibco), 5% fetal bovine serum (BioWest, Nuaillé, France), 1∶100 glutamine and penicillin/streptomycin (Gibco), 1∶100 non-essential amino acids (PAA Laboratories, Pasching, Austria), 1∶500 mercaptoethanol (Gibco) and 2000 Units mL^−1^ leukemia inhibitory factor (produced in-house).

### Transmission Electron Microscopy

For transmission electron microscopy (TEM), cells were fixed with 2.5% glutaraldehyde (Merck, Darmstadt, Germany) in 0.1 mol L^−1^ sodium cacodylate buffer, pH 7.4, post-fixed in 1% aqueous osmium tetroxide, dehydrated stepwise in graded ethanol series, and embedded in Epon 812 (Fluka, Buchs, Switzerland). Ultrathin sections (50 nm) prepared with ultramicrotome (EM UC6, Leica, Wetzlar, Germany) were stained with 1% uranyl acetate, 3% lead citrate, and examined using a Zeiss EM 109 electron microscope (Zeiss, Oberkochen, Germany).

### Ultrastructural Characterization of Mouse Embryos

Analysis focused on mitochondrial number, cristae and global morphology at 2-cell, 4-cell and morula stage ICSI and SCNT embryos, using TEM images (3000X). Specifically, total numbers of round or elongated mitochondria were counted in single sections of three different embryos, for each embryonic stage. Number of mitochondria with different cristae morphology (an indicator of maturation level) was determined. Three mitochondrial types were distinguished: 1) developed – mature, showing well-developed cristae; 2) medium developed – with less-developed, peripheral cristae; and 3) undeveloped – immature, with poorly developed or no distinguishable cristae, often containing membrane-bound vesicles.

### Cryo-immuno Electron Microscopy

Localization of EGFP was performed by cryo-immuno electron microscopy, as previously described [44]. Oocytes from 5 week-old superovulated B6C3F1 and mtGFP-Tg mice were fixed overnight (4°C), in 0.1 mol L^−1^ phosphate buffer (Merck), pH 7.4, with 2% paraformaldehyde and 0.2% glutaraldeyde (EM-grade, Science Services, Germany). Samples were washed and embedded in 12% gelatine (food-quality, Dr. Oetker, Germany). For orientated sectioning, samples were counterstained with 1% toluidin-blue in 2.3 mol L^−1^ sucrose (Baker Analyzed ACS) 10 min and infiltrated overnight in 2.3 mol L^−1^ sucrose for final cryoconservation. Destained blocks were mounted on pins and frozen (liquid nitrogen). 50 nm cryosections were cut at −110°C in a ultramictrome cryo-chamber (EM UC6, Leica). Sections were labeled with 1∶200 anti-GFP rabbit polyclonal antibody (A6455, Invitrogen, Germany), and detected by protein-A-gold conjugate (CMC, Utrecht, The Netherlands). Analyses were performed at 80 kV at the transmission microscope (Tecnai 12- biotwin, FEI, The Netherlands).

### Gene Expression Analysis

Complementary DNA synthesis from RNA (samples of pooled blastocysts, 96 hours after activation) was performed using a High Capacity cDNA Reverse Transcription Kit (Applied Biosystems, Foster City, CA, USA). Transcript levels were determined for two samples (over two independent experiments) in triplicate (technical replicates), in 20 µL reaction-mix/well consisting of cDNA (1∶10 dilution), primers, H_2_O and Power SybrGreen PCR Mastermix (Applied Biosystems), using ABI PRISM Sequence Detection System 7900ht (Applied Biosystems; primer sequences: Table 3). Run conditions were as described before [42]. Gene expression was normalized to an external reference, FLAG-Oct4 mRNA [45], added to the lysis buffer at sample collection time. Ct-values were obtained with SDS 2.2 (Applied Biosystems).

**Table 3:** Primer sequences for SYBR green PCR.

Gene	Mouse gene ID	Gene name	Primer sequence
*Actb*	11461	beta actin	5'-ACTGCCGCATCCTCTTCCTC-3'
			5'-CCGCTCGTTGCCAATAGTGA-3'
*Egfp*	n.a.	enhanced green fluorescent protein	5'-TGCAGTGCTTCAGCCGCTAC-3'
			5'-TCGCCCTCGAACTTCACCTC-3'
*Flag-Oct4*	n.a.	n.a.	5'-TGGACTACAAGGACGATGATG-3'
			5'-GGTGAGAAGGCGAAGTCTGA-3'
*Gapdh*	14433	glyceraldehyde-3-phosphate dehydrogenase	5'-CCAATGTGTCCGTCGTGGAT-3'
			5'-TGCCTGCTTCACCACCTTCT-3'
*Pou5f1*	18999	POU domain, class 5, transcription factor 1	5'-TGTTCCCGTCACTGCTCTGG-3'
			5'-TTGCCTTGGCTCACAGCATC-3'
*Tfam*	21780	transcription factor A, mitochondrial	5'-CATTTATGTATCTGAAAGCTTCC-3'
			5'-CTCTTCCCAAGACTTCATTTC-3'
*Vdac2*	22334	voltage-dependent anion channel 2	5'-GGCTCACGTATGTGCAGTTAC-3'
			5'-TATTGTAATCTCAAAGACCTCG-3'

Flag-Oct4 mRNA [45] was used as external reference. n.a., not applicable.

### EGFP Images Acquisition and Analysis

ICSI and SCNT blastocysts (resulting from manipulations of B6C3F1 recipient oocytes/ooplasts and sperm or cumulus cell nuclei of mtGFP-Tg) were imaged for GFP intensity. Individual embryos were placed in 5 µL embryo culture medium on a 50 mm thin-bottom plastic dish (Greiner Bio-One, Lumox hydrophilic dish; Frickenhausen, Germany), overlaid with mineral oil (M8410 Sigma). Images were captured on stage of an inverted microscope (Eclipse 2000-U; Nikon, Düsseldorf, Germany) coupled to a spinning disk confocal unit (Ultra View RS3; Perkin-Elmer LAS, Jügesheim, Germany). A Nikon CFI Plan Apochromat VC 40X oil immersion lens was used to convey laser excitation to the embryo (Argon/Krypton laser; Melles Griot, Albuquerque, New Mexico, USA). Optical sections for the whole embryo (2–5 µm interval) were captured using a Hamamatsu ORCA ER digital camera (Hamamatsu Photonics KK, Japan). For image analysis, maximum projections were analyzed with ImageJ. Region of interest (ROI) was drawn to include only pixels belonging to the embryo. Mean intensity or integrated density were determined.

### Cell Count

To determine the correlation between EGFP intensity and cell number, fertilized blastocysts (B6C3F1×mtGFP-Tg) were fixed in 1% paraformaldehyde (MP Biomedicals, Illkirch, France) in PBS (3–4 min, 30°C). To counterstain nuclei, after brief PBS wash embryos were placed individually in 5 µL drops of far-red fluorescent DNA dye Draq5 (Biostatus, Shepshed, UK), on a 50 mm thin-bottom plastic dish overlaid with mineral oil. Images were captured on the confocal unit as described above (Nikon CFI Plan Apochromat VC 40X oil immersion lens).

### Immunocytochemistry

Immunostaining was performed similarly to previously described [42]. Samples were fixed and permeabilized in 1% paraformaldehyde (MP Biomedicals) and Triton-X 100 (0.1% v/v) in PBS, 20 min 30°C. Samples were briefly washed in PB-T (PBS containing 0.1% Tween 20), and incubated at 4°C in blocking solution containing 2% BSA (w/v), 2% glycine and 5% donkey serum (previously inactivated through heat treatment, 55°C 30 min). Depending on the experiment, samples were transferred into primary antibody in a 1∶4 dilution of blocking solution, and incubated overnight. Primary antibodies were: anti-GFP rabbit polyclonal (Invitrogen A6455), 1∶200; anti-Actb (20–33) rabbit polyclonal (Sigma A5060), 1∶200; anti-Gapdh (6C5) mouse monoclonal (Ambion AM4300), 4 µg mL^-1^. After two washes in PB-T, specimens were incubated in 1∶4 dilution of blocking solution containing secondary antibody, 30°C. Secondary antibodies used were (1∶2000 dilution, 30°C 1 hour): goat anti-rabbit antibody coupled to Alexa fluorophore 647 (GFP); donkey anti-rabbit antibody coupled to Alexa fluorophore 488 (Actb); donkey anti-mouse antibody coupled to Alexa fluorophore 647 (Gapdh). Samples were washed twice in PB-T and placed in PBS drops on 50 mm thin-bottom plastic dish overlaid with mineral oil. Images were captured and analyzed as described above, using a Nikon CFI Plan Fluor 20X (EGFP) or Nikon CFI Plan Apochromat VC 40X oil immersion lense (Gapdh and Actb).

### Microarray Analysis

Microarray analysis was performed on MIAME compliant data previously obtained from our laboratory, made accessible in the public microarray repository ArrayExpress (http://www.ebi.ac.uk/arrayexpress) with accession number E-TABM-1182. Briefly, to obtain these data, Illumina bead chip hybridizations were performed on embryos at the 8-cell stage (56 hours after activation), lysed and frozen at -80°C until processing. Two independent biological replicates were collected. Total RNA was isolated using the MicroRNeasy Kit (Qiagen, Hilden, Germany), and a two-round linear amplification protocol using a linear amplification kit (TargetAmp aRNA amplification kit, Epicentre Biotechnologies, Madison, WI, U.S.A.) was adopted to generate biotin-labeled cRNA. 1.5 µg of which was used for each hybridization reaction. cRNA samples were hybridised onto Illumina mouse 8 BeadChips. Washing, Cy3-streptavidin staining, and scanning were performed on the Illumina iScan (Illumina, San Diego, CA, U.S.A.) platform using reagents and protocols from the manufacturer. Expression data analysis was carried out using Bioconductor [46], an open source software package based on the R programming language (R Development Core Team, 2011). Bead summary data (not normalized, including regular probe profile and control probe profile) and associated probe annotation were taken from Illumina GenomeStudio. Raw data were background-subtracted, transformed for variance stabilization and normalized based on robust splines, using the lumi R package [47]. Normalized data were then filtered for significant expression on the basis of negative control beads. Fold changes and standard errors were estimated after fitting a linear model for each gene using the limma R package [48]. Probes with at least one log2-fold change were selected as differentially expressed and further analysed for enrichment in Gene Ontology (GO) biological process terms; signals considered were in absolute terms, i.e. on both the positive – upregulation – and the negative– downregulation – spectrum of expression. Significance for each individual GO term was computed using the topGO R package by applying Fisher’s exact test with parent-child algorithm [49], which takes the GO dependencies into account. Significance testing was performed with a p-value truncated at 0.01.

### ATP and ADP

ATP levels in SCNT and ICSI embryos were determined using an ATP-dependent luciferase-luciferin reaction system, as described before [42]. Five to 8 embryos were collected in 200 µL milli-Q H_2_O, vortexed (1 min), heated (95°C 20 min), and stored (−80°C). Samples were incubated in 1 mol L^−1^ MgCl_2_, 2 mol L^−1^ KCl and 0.2 mol L^−1^ phosphoenolpyruvate, with and without pyruvate kinase (which converts ADP to ATP), 30°C 15 min. Luciferase-luciferin mix (ENLITEN; Promega, Mannheim, Germany) was added, and light emission detected by single photon counter luminometer (Tecan GENios Pro; Tecan Group Ltd, Männedorf, Switzerland). ATP levels were extrapolated from a calibration curve of known ATP concentrations; ADP was calculated from a linear equation obtained by subtracting two calibration curves: [ATP+ADP] - [ATP].

### Hydrogen Peroxide

SCNT or ICSI blastocysts were incubated 15 min in embryo culture medium containing 10 µmol L^−1^ DCHFDA (2′,7′-dichlorodihydrofluorescein diacetate; Fluka), for hydrogen peroxide and related peroxides determination. Embryos were washed in culture medium and quickly imaged in individual drops, as described for acquisition of EGFP images. Images were taken at 5 time points, 10 sec apart (200–400 ms exposure, 2x2), allowing fluorescence signal increase due to ROS (induced by laser exposure), to confirm probe specificity. Images from the first time point were analyzed, as described above; ROI was taken for the whole embryo.

### Intracellular Glutathione

Intracellular reduced glutathione was assess by imaging the fluorescent adduct formed with monochlorobimane (MCB), as described previously [50]. Embryos were imaged (exc: 360 nm; em: 470 nm longpass) and then exposed to medium containing 12.5 µmol L^−1^ MCB. Increase in fluorescence was captured (Leica CTR 6500 microscope), and quantification of fluorescence determined by image analysis (ImageJ).

### Mitochondrial Membrane Potential and Cytosolic Calcium

Embryos were incubated (37°C 5% CO_2_) in embryo culture medium containing 30 nmol L^−1^ tetramethylrhodamine methyl ester (TMRM; Invitrogen) for mitochondrial membrane potential, and 2 µmol L^−1^ Fura-2 AM (Invitrogen) for Ca^2+^ determination. Embryos were placed in HCZB drops containing TMRM and Fura-2 AM, on a glass slide, rapidly but carefully covered with coverslip to keep embryos in place (with excess media) and placed on a Leica CTR 6500 microscope stage (30°C). Total incubation time (i.e. until imaging start) was 1 h 30 min for TMRM, and 35 min for Fura-2 AM. Embryos were imaged 10 min, before HCZB containing 1 µmol L^−1^.

carbonyl cyanide 4-(trifluoromethoxy)phenylhydrazone (FCCP; Fluka) was added, to determine basal mitochondrial membrane potential and maximum cytosolic Ca^2+^ levels. Excitation light (TMRM, 595 nm; Fura-2 AM 400 nm) was delivered by a monochromator, while emission was collected with filters (TMRM, 645/75 bandpass; Fura-2 AM, 470/40 bandpass) to a Hamamatsu camera. Integrated density was determined for ROI drawn to include pixels of the embryo. For membrane potential, fluorescence is expressed as signal initially measured (i.e. before FCCP) minus basal signal (i.e. after FCCP).
